# Young Mothers

**Published:** 1873-07

**Authors:** 


### YOUNG MOTHERS.

It seems beyond dispute that a mother casts the die of her child's destiny—physical, and moral, and emotional—by the method of her life for the year preceding birth, and during that year she has it in her power to decide whether the child shall be safely born, easily born, painlessly born and healthfully born, or not. The examples given in "Health at Home" on this subject are conclusive. A good constitution and a high moral and religious sentiment are given to the child through the mother, and are determined by her obedience or disobedience of the laws of life. Right nursery training does a very great deal in this direction, and may modify it afterwards, but it is antenatal training that gives the bent, that moulds the constitution and the character; if, during the forming stages of existence, while yet the breath of life has not been drawn, the mother is weakly, and fretful, and complaining, and foreboding, or gives loose to appetite and indulgence, and the passions of our nature, so will be the child that is to be born into the world. These suggestions merit the attention of all thoughtful men and women; in fact, it may be urged as a Christian duty to bestow that attention to so grave and momentous a subject.

Certain it is that the aims, and ambitions, and aspirations of the mothers of a Bonaparte, a Henry Clay, a Horace Greeley—yes, of Samuel, and Samson, and Solomon, and Daniel, and John—founded and developed, and then lived again in the illustrious names which this page records. It may be noted here that all these mothers most earnestly desired children—a thousand times more motherly women, more womanly women than that increasing number now found, who, in various ways, are aiming and attempting to invalidate the injunction of Divinity in the words, "Multiply and replenish."

